# Role of Post-Transcriptional Regulation in Learning and Memory in Mammals

**DOI:** 10.3390/genes15030337

**Published:** 2024-03-05

**Authors:** Carlo Maria Di Liegro, Gabriella Schiera, Giuseppe Schirò, Italia Di Liegro

**Affiliations:** 1 Department of Biological, Chemical and Pharmaceutical Sciences and Technologies, University of Palermo, 90128 Palermo, Italy; carlomaria.diliegro@unipa.it (C.M.D.L.); gabriella.schiera@unipa.it (G.S.); 2 Department of Biomedicine, Neurosciences and Advanced Diagnostics, University of Palermo, 90127 Palermo, Italy; giuseppeschiro1994@gmail.com; 3 Neurology and Multiple Sclerosis Center, Unità Operativa Complessa (UOC), Foundation Institute “G. Giglio”, 90015 Cefalù, Italy

**Keywords:** post-transcriptional regulation, epigenetic control, mRNA localization, RNA-binding proteins (RBPs), non-coding RNAs, mRNA metabolism, mRNA transport

## Abstract

After many decades, during which most molecular studies on the regulation of gene expression focused on transcriptional events, it was realized that post-transcriptional control was equally important in order to determine where and when specific proteins were to be synthesized. Translational regulation is of the most importance in the brain, where all the steps of mRNA maturation, transport to different regions of the cells and actual expression, in response to specific signals, constitute the molecular basis for neuronal plasticity and, as a consequence, for structural stabilization/modification of synapses; notably, these latter events are fundamental for the highest brain functions, such as learning and memory, and are characterized by long-term potentiation (LTP) of specific synapses. Here, we will discuss the molecular bases of these fundamental events by considering both the role of RNA-binding proteins (RBPs) and the effects of non-coding RNAs involved in controlling splicing, editing, stability and translation of mRNAs. Importantly, it has also been found that dysregulation of mRNA metabolism/localization is involved in many pathological conditions, arising either during brain development or in the adult nervous system.

## 1. Introduction

Nowadays, the hypothesis of a primeval word in which what we know as messenger RNA (mRNA) was also the only existent genetic material [1,2,3,4,5] is largely accepted, and it has also been suggested that its ability to interact with both proteins and different kinds of non-coding RNAs is indeed a remnant of that “RNA world” [6,7].

In those primordial times, RNA–protein interactions were probably fundamental, both for RNA replication and for its translation into what were probably very simple sequences of amino acids. It has also been hypothesized that such short peptides, with their simple composition, were able to form some sort of aggregate, which allowed for RNA concentration and the potentiation of its intrinsic enzymatic activity [8], thus enabling replication. Similar properties are found today in amyloid-forming proteins that contain, indeed, intrinsically disordered regions (IDRs), probably involved in the formation of membrane-less structures, that are able to interact with RNA. In other words, the ancient “RNA world” might have also been a sort of “amyloid world” [9,10,11,12,13], the heirs of which are the amyloid-forming proteins observed in many neurological diseases as well as the many proteins that, in physiological conditions, can form, with different classes of RNA, granules [14,15,16] that allow maturation, transport and regulated translation of mRNAs. With the advent of DNA as a more stable genetic material, as well as with the evolution of the complex genetic regulatory system based on the control of chromatin structure, RNA–protein [17,18] and RNA–RNA interactions [19] should have been conserved, thus allowing a much more precise system of regulation, based on the control of RNA metabolism, localization and regulated translation, in response to specific signals. Moreover, among RBPs, an important role has been attributed to proteins with prion-like domains, such as the Cytoplasmic Polyadenylation Element Binding (CPEB) protein [20,21,22]. Interestingly, even some of the nucleoporins, the proteins that constitute the nuclear pores through which the RNA–protein complexes make their way to the cytoplasm, have been found to have amyloid properties [23].

**Figure 1:**
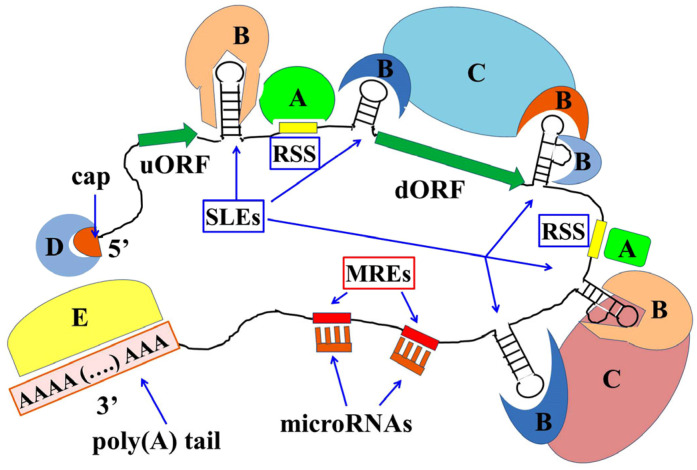
**Schematic drawing of mRNA structure and its interactions with both RBPs and miRNAs**. The mRNA molecule exhibits a cap structure at its 5′-end and a poly(A) tail at its 3′-end. Any mRNA contains at least one open reading frame (**ORF**) that will be translated into protein, and sometimes more than one open reading frame is present; in this latter case, the ORF are called “upstream” (**uORF**) and “downstream” (**dORF**) ORF, respectively. As indicated in the picture, recognition simple sequences (**RSS**) as well as stem-loop elements (**SLEs**) are present for interactions with RBPs. Short element sequences called miRNA recognition elements (**MREs**) are also present. Among the proteins able to interact with mRNA, some (**A** in the picture) bind to simple sequence elements, while others (**B** in the picture) recognize and bind stem-loop elements. Finally, some proteins (**C** in the picture) cooperate in the formation of RNPs by binding to the proteins that directly bind to mRNA. Moreover, other proteins bind to the 5’-end (**D**) or to the 3’-ens (**E**) of the mRNA.

The ability of mRNAs to interact with both RBPs and ncRNAs depends on the presence of simple nucleotide sequence elements, recognized and bound by proteins (simple sequence elements: SSE), and by complementary sequences recognized by microRNAs (miRNA recognition elements: MRE); moreover, they also contain secondary/tertiary structure elements (stem loop structures: SLS), that are recognized by other RBPs. In addition, specific proteins bind their cap structure, at the 5′-end, as well as the poly(A) tail, at the 3′-end (Figure 1). Interestingly, the interaction between the cap-binding proteins and the poly(A)-binding ones can allow circularization of the mRNAs that, thus, depending on the context, result in even more stabilization (i.e., their ends are not accessible to nucleases) or easier translation (i.e., ribosomes that complete translation can immediately find the 5′-end to start again).

All the regulatory events mentioned above are very much used in the nervous system [24,25,26,27,28], first of all during development, in order to generate the asymmetry that characterizes its structure and function, but also in the adult brain, which allows for the stabilization and modification of the synapse structure in response to neurotransmission, thus also determining the highest brain cognitive functions, such as learning and memory [20,21,22,29,30].

Indeed, in the nervous system, it has been found that the actual activity across synapses modifies the responses of the involved neuronal elements in a neurotransmission-dependent manner, thus determining a long-term potentiation (LTP) or a long-term depression (LTD) of the connection between the pre- and the post-synaptic elements. The mechanisms underlying LTP are fundamental for the highest cognitive functions, such as the processes of learning and memory. Among other things, a correlation has been reported between the synaptic strength and the volume of the dendritic spines that constitute the post-synaptic elements [31,32,33,34,35,36]. Modifications of the dendritic spines are, in turn, bound to the remodeling of the actin cytoskeleton, a complex event which also depends on the modification of the number and activity of actin-binding proteins able to regulate G-actin polymerization. Interestingly, the trans-membrane coupling among actin filaments and the extracellular matrix also seems to perform a fundamental function in LTP, thus suggesting a cooperation among molecular modifications and changes in their ability to exert mechanical forces [37]. It is also important to consider that learning and memory are, for most, bound to neurotransmission from glutamatergic neurons; in this case, due to the involvement of the N-methyl-D-aspartate-type glutamate receptors (NMDARs), post-synaptic elements undergo a significant afflux of calcium ions, which can bind and activate the Ca^2+^/calmodulin-dependent protein kinase II (CaMKII). It has indeed been found that the generation of LTP is also CaMKII-dependent [36,38].

Although these events have been clearly recognized, the precise molecular factors involved are still a matter of intense study. For example, some laboratories are taking advantage of the possibility of using human-derived pluripotent stem cells in order to obtain organoids able to function as in vitro models of brain development and functions [39]. The interest devoted to LTP generation is also due to observations that indicate there are age-related differences in the ability to generate LTP, as well as variations in LTP generation, and, as a consequence, in learning and memory activities experienced in many neurodegenerative diseases [40].

One further aspect that has now been largely accepted as fundamental for synapse plasticity is the ability of both the pre- and post-synaptic elements to synthesize new proteins in response to neurotransmission, and this capacity clearly depends on the localized and regulated translation of mRNAs encoding proteins involved in the above mentioned structural/functional adaptations of the synapses. As we will discuss below, these events are controlled by epigenetic factors, such as modifications in different aspects of mRNA metabolism, and including localized splicing of the molecules and modification of specific nucleotides.

Here, we will discuss the known mechanisms at the basis of the cited events, in which both RNA-binding proteins (RBPs) and non-coding RNAs (ncRNAs) are involved, specifically focusing on the processes of learning and memory.

## 2. Post-Transcriptional Regulation and Synaptic Plasticity during the Nervous System’s Development

The formation of synapses is, of course, an event of central importance during neuronal differentiation and, in general, during the maturation of the cerebral network. As mentioned above, both RBPs and ncRNAs are involved in these processes; some of these regulatory factors are then also active in the adult brain, where they regulate adult synapse plasticity. As an example, the human antigen D (HuD), also known as ELAV (Embryonic Lethal, Abnormal Vision, *Drosophila*)-like protein 4 (ELAV4), is a neuronal-specific RBP, involved in gene expression control both during brain development and in the adult brain. Thanks to three RNA recognition motifs (RRMs), it is able to bind and stabilize mRNAs that have in their 3′-untranslated region (3′UTR) adenine- and uridine-rich instability-conferring elements (AREs) [30,41,42,43,44]. HuD target mRNAs encode a variety of proteins [45,46,47], among which is the growth-associated protein-43 (GAP-43) [48], as well as mRNAs that encode neurotrophic factors, such as nerve growth factor (NGF), brain-derived neurotrophic factor (BDNF) and neurotrophin (NT)-3 [49]. Its activity and distribution are regulated, in turn, by a neuronal protein kinase C (PKC), that thus plays a central role in neuronal maturation and in synaptogenesis [49]. Similarly, ncRNAs, and in particular microRNAs (miRNAs), have a role both during development and in the adult brain [50,51,52,53,54,55,56,57]. As an example, miRNA-124 has been reported to play a role as a promoter of neurogenesis, but also in mature, post-mitotic neurons, where it seems to be involved in synapse plasticity and hence in learning and memory [58]. We will now discuss how regulation of mRNA metabolism and localization controls neuronal differentiation and the formation of the synaptic web that characterizes the mature brain.

### 2.1. Prelocalization of mRNAs and the Role of RNA-Binding Proteins and miRNAs in Normal Development

During brain development, post-transcriptional regulation of gene expression plays a central role in all the cell types that will contribute to the complex structure of the nervous system. In particular, different steps of mRNA metabolism are involved in this sort of regulation, during neuronal maturation, starting from completion of the “heterogeneous nuclear” RNA (hnRNA) transcription up to the arrival to the final cytoplasmic destination of the mature form of the messenger. As mentioned above, in order to obtain a functional mRNA, localized at the right place and ready to be translated as a response to specific signals, and to neurotransmission in particular, many RBPs, different enzymes and ncRNAs are required, which should be, in turn, synthesized and localized at the right moment and ready to interact, also in the right relative amounts, with the neosynthesized mRNA.

#### 2.1.1. RNA Processing and Localization

One of the processes involved in mRNA maturation is represented by splicing, which often undergoes tissue- or even cell-specific “alternatives”. This latter possibility allows for the production of a high number of cell-specific proteins, even with a limited number of expressed genes. Actually, the splicing process is quite complex, and requires both RNA nucleotide sequences and specific sets of proteins, as well as small RNAs that together form the so-called small nuclear ribonucleoproteins (RNPs).

In particular, the role of some of the proteins included in these complexes is to induce the right hnRNA conformation that allows intron removal, and the choice of the exons to be included in the mature mRNA [59,60]. When speaking of alternative splicing, it is important to mention that its function is not only to allow the formation of alternative proteins but also to allow the inclusion of specific regulatory sequences into the mRNA, which will then affect both messenger localization and translation. Among the best-known examples of this effect are mRNAs encoding BDNF and the Calcium/Calmodulin-Stimulated Protein Kinase II (CaMKII); in these molecules, alternative splicing allows inclusion into the mRNA 3′UTR of a dendritic targeting element (DTE) [26]. Interestingly, alternative splicing of the CAMKII mRNA can also take place directly in dendrites [7,61,62]. Another notable example of a collection of protein isoforms obtained thanks to alternative splicing in neurons is represented by neurexins, which are able to act as receptors for different proteins and have been suggested to be involved in the structural and functional organization of the presynaptic region, as well as, in turn, in determining the typology of single neurons [63,64,65].

Other fundamental mechanisms that guide neurons during development are the localization of the mRNAs, and the regulation of their stability and translatability [7,66]. The structure of the mRNA 3′UTR and, in particular, the poly(A) tail, has great importance in a messenger’s destiny because it has a role in different moments of its life cycle. The poly-A tail is indeed needed during export: it may enhance stability and creates links to proteins controlling the efficiency of translation [67,68,69,70]. Notably, it is now clear that cell-specific alternative polyadenylation (APA) profiles do exist, and that, for example, proliferative cells often have mRNAs with shorter 3′UTRs [71], whereas terminally differentiated cells, such as neurons, often show longer ones [72]. Interestingly, there is a transition towards mRNAs with longer 3′UTR during neuronal differentiation [73]. As a consequence, modulation of poly(A) tail length and APA function, together with the cytoplasmic poly(A) binding protein (PABPC), might be part of a process that allows coordination of the expression of a collection of mRNAs during differentiation [70,74].

#### 2.1.2. The Developmental Impact of RNA-Binding Proteins

After maturation of hnRNA, some RBPs—which are important for splicing regulation, detach from mRNA—while other RBPs—important for interactions with the nuclear pore proteins and for export from the nucleus, localization and translation of mRNA-containing RNPs—remain bound or bind “ex novo” to it [75,76]. This is of the most importance to determine subcellular distribution of mRNA, a process under dynamic regulation [77]. For example, as demonstrated by studies on mice, during vertebrate neurogenesis, genes encoding neuronal members of the Embryonic Lethal Abnormal Vision (nELAVL) protein family, first described in Drosophila, are expressed in a specific spatial and temporal way [78]. In particular, the role of Elavl2 has been studied, using RNA-mediated gene silencing (RNAi), in Apis mellifera (honeybee); this organism cannot be considered a general model organism, since its genome contains only one gene for Elavl proteins (Elavl2). However, ELAVL2 is similar to the proteins found in neurons. Moreover, even if, in this organism, there is only one gene for these proteins, a sort of compensation does exist, due to a complex pattern of alternative splicing, which allows for the production of a high number of isoforms. On the basis of behavioral studies, it has been suggested that, in honeybees, Elavl2 is involved in the formation of associative memory [79].

An interesting group of RBPs, clearly involved in the first steps of mRNA metabolism but also in its localization and utilization in the periphery, is formed by some of the heterogeneous nuclear RNA-binding proteins (HnRNPs). HnRNP A/B has been clearly identified in the developing olfactory sensory neurons (OSNs), where it binds mRNAs, thus allowing their expression in the axonal ends. The regulation of this process has been found to be essential for OSN maturation, as well as for the generation of odor perception abilities [80].

Notably, the general morphology of the migrating neurons is also finely regulated by mechanisms which depend on the localized and time-specific translation of many different mRNAs. In 2015, Murn et al. [81] reported, for example, that, in the mouse embryo, depletion of an RBP, called Unkempt, interfered with the shaping of neurons, while, on the other hand, its expression in cells that normally do not produce it induced them to take a neuronal-like shape. In particular, the Authors proposed that Unkempt, a zinc finger-containing protein, might have a coordination role that is also based on the regulation of expression of other RBPs (such as HnRNPs, Staufen proteins and ELAV proteins), each of which has its own mRNA targets [81]. More recently, it has been found that Unkempt is clearly involved in cognitive flexibility [82]; moreover, it is a target of the kinase known as mammalian target of rapamycin (mTOR), and this mTOR-dependent phosphorylation is fundamental for the regulation of cell morphology by Unkempt [83].

A role in the elaboration of neuronal cytoarchitecture has also been attributed to the proteins Pumilio homolog 2 (Pum2) and TAR DNA-binding protein 43 (TDP-43); these proteins are indeed able to regulate, at the post-transcriptional level, the expression of mRNAs encoding proteins involved in determining the specific organization of the cerebral structures, including the neocortex [84].

Aside from the RBPs clearly involved in morphogenic processes, other proteins in the family control the balance between the proliferation and differentiation of neural progenitors; for example, it has been recently reported that the polyglutamine binding protein 1 (PQBP1) controls the alternative splicing of the mRNA encoding NUMB, a protein involved in endocytosis but also in the maintenance of the neural progenitors (also known as radial glial cells). Interestingly, it has been found that the inclusion of exon 9, promoted by PQBP1, during the alternative splicing events generates a NUMB isoform that stimulates progenitor proliferation [85]. Similarly, the proteins belonging to the RNA-binding “feminizing locus on X” (Rbfox) regulate the splicing of different mRNAs, among which are some of the encoding proteins needed for the cytoskeleton, as well as for synaptic formation and functioning [86]. Actually, most RNAs encoding proteins involved in the formation of the axon segment, and in initiating action potential activity, undergo splicing mediated by Rbfox proteins [87]. Further examples of RBPs involved in splicing are the neuro-oncological ventral antigens 1 and 2 (NOVA1 and NOVA2). By controlling maturation of the mRNAs encoding the Netrin/Deleted in colorectal cancer (Netrin/DCC) receptor and the SLIT/ Roundabout (ROBO) cell signaling protein, they regulate fundamental processes, such as axonal guidance and cortical layer development [66,88].

As we will discuss below, another protein of interest is the fragile X mental retardation protein (FMRP), an RBP that regulates localization and translation of many mRNAs [89]. Although this protein has been essentially considered a translational inhibitor [90,91], it has been recently found that it can enhance translation of proteins essential for normal neurite outgrowth [92].

#### 2.1.3. The Role of miRNAs in Brain Development

As mentioned above, besides RBPs, many studies have also evidenced the involvement of microRNAs in the regulation of developmental/differentiation processes that allow for the formation of the different brain structures. For example, members of the miR-17 family inhibit the differentiation of NPCs into astrocytes and promote their proliferation [93]. Other miRNAs, like miR-124, instead have an impact on the formation and growth of neural projections [94]; included among its targets are mRNAs encoding repressors of neuronal differentiation, such as Ras homolog family member G (RhoG), paired box gene 3 (PAX3) and BRG1-associated factor 53A (BAF53a) [95]. On the other hand, miR-124 controls many steps of neurogenesis, assembly of neuronal networks and synaptic plasticity [96]. Similarly, miR-128 has been implicated in a variety of developmental aspects, such as neuronal migration and progenitor cell fate determination [88,97]. In other words, brain/neuronal-specific miRNAs are involved in the regulation of almost all the stages of cell lineage development, from the initial proliferation of NPCs to the establishment of synapses and synaptic plasticity [98]. As expected, production of miRNAs also undergoes regulation; for example, loss of miR-107 causes upregulation of the Dicer enzyme and, in turn, of miR-9; these modifications led to abnormal neurogenesis during zebrafish hindbrain development, suggesting that miR-107 has a modulatory and fundamental role in the production of pro-neurogenic miRNAs [99]. Actually, it is important to consider that miRNAs probably form networks with overlapping functions in order to coordinate expression of their targets [97].

In addition, miRNAs also play a central role in glial development and function, thus also having an indirect impact on neurons, given the continuous cross-talk among these cells (see below). For example, some miRNAs, such as miR-219 and miR-338, are oligodendrocyte-specific and play crucial roles in driving oligodendroglia development and myelin production [100,101,102].

#### 2.1.4. The Developmental Role of mRNA Modifications

Notably, modification of mRNA bases is also important in order to determine the messenger’s fate and, as a consequence, for the regulation of development and/or differentiation capability of nerve cells. It has been found, for example, that mRNAs from genes involved in neuronal differentiation are enriched, in the developing cortex, with N6-methyl-adenosine (m6A). Moreover, it has been found that these mRNAs exhibit highly reduced half-life compared to mRNAs without m6A modification. Thus, probably, m6A can destabilize target mRNAs involved in processes such as self-renewal or differentiation of neural progenitors in the cortex, and this ability supports the rapid transition in gene expression required for the progression of neurogenesis [103]. Moreover, a recent study reported that nascent, m6A-tagged transcripts can recruit themselves to the modified histone H3K9me2, in chromatin, and the lysine demethylase 3B (KDM3B) via the YTH domain-containing protein 1 (YTHDC1), thus allowing for the removal of the repressive histone mark H3K9me2 and, in turn, the stimulation of transcription [104]. All these observations, involving both epigenetic and epitranscriptomic events, are thus very important for the right progression of embryonic neurogenesis [105].

#### 2.1.5. Prelocalization of mRNAs and the Role of RNA-Binding Proteins in Developmental Pathologies

Dysfunction of RBPs and interruption of their role in the control of RNA prelocalization can lead to the onset of some developmental pathologies, one of which is fragile X syndrome, the most frequent cause of hereditary mental retardation. The syndrome is caused by different mutations, such as the trinucleotide CGG repeat expansion in the 5′-UTR of the gene, which causes hypermethylation of the promoter, and downregulation (or even complete absence) of the fragile X mental retardation protein (FMRP). As mentioned, FMRP is an RBP normally expressed at high frequency in the brain, where it regulates expression of proteins related to maturation and development. In particular, it regulates localization and translation of messengers. Actually, FMRP binds to many target mRNAs and regulates their localization at the level of dendritic terminals, as well as in axons; many FMRP-regulated RNAs encode for axonal proteins [106]. For example, delivery of miR-181d by FMRP-containing granules to the axonal terminal of primary sensory neurons affects axonal elongation by targeting the microtubule associated protein 1B (Map1b) and calmodulin 1 (Calm1) [107]. The location of FMRP at the axonal level appears to be regulated by transport along microtubules. It seems, indeed, that, in order to reach the axonal terminals, FMRP associates with endolysosomal organelles. Interestingly, it should also be transported in a retrograde manner by the dynein light chain roadblock 1 (Dynlrb1), a subunit of the dynein complex. Silencing Dyn1rb1 causes accumulation of FMRP granules at the axonal terminal with a subsequent reduction in Calm1 translation and, in the end, sensory neuron degeneration [108]. Although it has been shown that, during brain development, FMRP granules also accumulate into F-actin-rich compartments, disruption of F-actin does not result in reduced levels of FMRP at the dendritic level, unlike the destruction of microtubules. In fact, as shown in a model of hippocampal neurons, it would seem that the localization of FMRP-associated mRNA granules requires the presence of microtubules [109].

Alterations of RNA metabolism by mutated FMRP may also be associated with the onset of autism spectrum disorders (ASD). In particular, FMRP seems to suppress the translation of proteins which are part of the trans-synaptic neurexin/neuroligin complex, such as Nrxn1, Nlgn3 and Nlgn4. However, it has also been reported that Nlgn1, Nlgn2 and Nlgn3 are downregulated by FMRP knockdown in cultured hippocampal neurons [110].

## 3. Post-Transcriptional Regulation and Synaptic Plasticity in the Adult Brain: Learning and Memory

Notably, many of the mechanisms, and the regulatory factors, involved in generating the asymmetry of the nervous system, as well as the highly complex network of nerve cells, during development are still active in the adult brain, where they ensure synaptic plasticity (i.e., modification of synaptic strength), and hence higher functions underlying nervous system adaptation, related to learning and memory. For example, it has been found that the RNA-binding protein HuD has a role in learning and memory in adult mice, and, in particular, that it specifically ensures the reinstatement of a response to food rewards [111].

**Figure 2:**
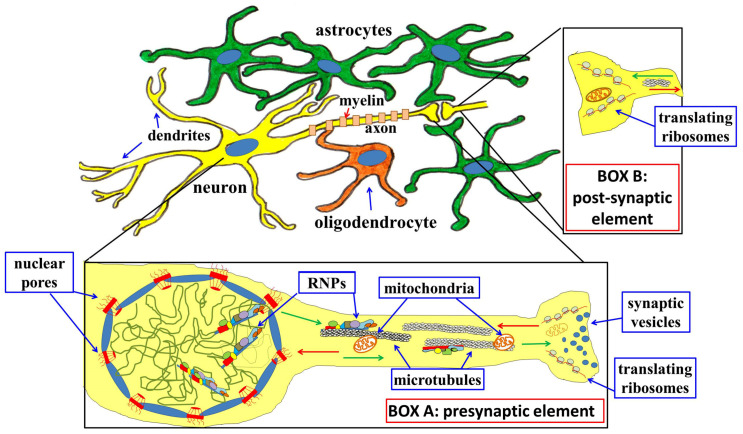
Schematic drawing of a neuron (yellow) and of one of its synapses. Around it, many astrocytes (green) have been outlined. Very close to it, an oligodendrocyte (dark orange) is also visible; this latter cell is responsible for myelination of the neuronal axon. Boxes A and B show enlarged views of the pre- (Box A) and post-synaptic (Box B) elements, respectively. **Box A**: mRNAs are transcribed in the nucleus, and immediately start interacting with many different kinds of RNA-binding proteins, involved in its maturation; the mature RNA-protein complexes (RNPs) are then allowed, thanks to interaction with components of the nuclear pores, to exit the nucleus. In the cytoplasm, RNPs interact with motor proteins that also interact with microtubules, thus allowing for the delivery of RNPs to different parts of the cells. In the figure, only RNPs directed to the axon have been indicated: green arrows indicate this anterograde traffic of RNPs. Together with these, many other objects, among which are synaptic vesicles and mitochondria, are transported by microtubules. RNPs will localize to synapses, where they will be modified in response to specific signals that will allow translation by localized ribosomes. Among the newly synthesized proteins, some will come back to the nucleus by retrograde transport (red arrows). **Box B**: neurotransmission will also activate pre-localized mRNA translation in the post-synaptic element; some of the proteins thus synthesized will change the structure/strength of the synapse, while others will be transported to the nucleus (red arrow). In both the pre- and the post-synaptic elements, proteins that reach the nucleus can contribute to changing the chromatin structure and transcriptional activity.

In general terms, the main regulatory processes rely on mRNA localization at synapses, and on their translation, sometimes even preceded by final splicing events, and/or base modifications, somehow coupled to neurotransmission [Figure 2].

As during development, both RBPs [7,112] and ncRNAs [53,54,55,56,113,114,115,116] are responsible for all the different steps of these regulatory events. Among these latter molecules, some small nucleolar RNAs (snoRNAs) also seem to play a role in learning and memory [117]. Moreover, neuronal activity itself has been found to regulate the concentration of many miRNAs; this effect seems to be due to cleavage and activation of the enzyme Dicer, an RNAse III involved in miRNA maturation that also localizes to postsynaptic densities (PSD) [118]. Moreover, further transcription of microRNA precursors can be controlled by neuronal activity-induced activation of the cyclic AMP element-binding protein (CREB) [118].

A further remarkable aspect of mRNA translation at the synapses is the fact that, as discussed below, some of these RNAs encode proteins that are then transported back to the nucleus, where they can bind to chromatin, thus modifying its structure and allowing for the modification of its transcriptional potential.

### 3.1. Prelocalized mRNAs and RNA-Binding Proteins in the Normal Adult Brain

Brain cells, and especially neurons, are all characterized by unequal distribution in different cell regions of organelles, but also by a variety of proteins. Concerning these latter molecules, from an energetic point of view, it is not useful to synthesize every single protein in the cell body, and then to transport it to the different peripheries; it is more convenient to organize complexes/granules which contain the corresponding mRNA, together with a series of other functionally related mRNAs, and then to transport these complexes to the different cell regions where they can be stored up to the moment in which translation is required. In other words, energy is thus only required to transport mRNAs that can then be translated many times, giving rise to many copies of the same proteins. As an exception to this general observation, it has been reported that the mRNA encoding the AMPA glutamate receptor A2 subunit (GluA2) is translated in the cell body and that concentration of GluA2 at the level of synapses is mainly regulated at the level of protein trafficking [119]; translation of this mRNA in the cell body is regulated by the inhibitory miR-124, which binds to its 3′UTR [119]. Nucleotide sequences present in the 3′UTR of mRNAs are indeed the usual targets for the binding of regulatory miRNAs [115]. More recently, the role of miR-124 in learning and memory has been further confirmed [120].

The mechanisms underlying the specific localization of mRNAs (and, of course, of the RNPs that contain them) are of the most importance; many observations also suggest that there are differences in the times needed for transporting different RNPs as well as in their average half-life [121]. Actually, when we consider the time necessary to deliver proteins to synapses, it becomes even more evident why prelocalization of mRNAs and localized synthesis of the corresponding proteins is essential to allow rapid modification of the function/structure of synapses, depending on neurotransmission.

Localized protein synthesis also requires the presence of chaperones to allow the correct folding of the newly synthesized molecules. Recently, it has been shown that mRNAs encoding chaperones also localize to dendrites; moreover, these mRNAs also increase upon stress, thanks to further microtubule-mediated transport [122]. Given the importance of proteostasis at the level of synapses, it is not surprising that protein degradation also has a role in controlling the concentration of proteins involved in synaptic plasticity. For example, it has been suggested that neuronal receptor 2 for apolipoprotein E (ApoER2) could be involved in the control of dendritic spine morphogenesis and, hence, in learning and memory [123]; interestingly, its concentration at the synapses is regulated by an E3 ligase, known as an inducible degrader of the LDL Receptor (IDOL) that, by ubiquitinating it, induces its proteasomal degradation [124].

Among the RBPs involved in the post-transcriptional regulation of mRNA in the nervous system, the most studied have been the already mentioned proteins FMRP, CPEB and NOVA [125]. In addition to these proteins, a large number of other RBPs have been discovered and found to function in one of the different regulatory steps that ensure the right localization and translational activation of mRNAs encoding proteins able to modify synapse strength and, hence, learning and memory; this observation suggests combinatorial activity among all these factors that allows a fine tuning of mRNA expression at the synapses. For example, the already mentioned protein known as GAP-43 is a presynaptic phosphoprotein that probably functions as a coordinating center for a large group of proteins and kinases involved in axonal structure and function, as well as in synapse plasticity control [126,127]. As long as it concerns the CPEB protein, its importance at the level of synapses has also been confirmed by recent experiments in Drosophila that demonstrated that, when the 3′UTR of its mRNA is deleted, the protein (known as Orb2 in this organism) is no longer specifically localized and, as a result, a clear deficit is found in the process of long-term memory acquisition [128,129]. Recently, it has been suggested that the critical role of CPEB proteins in translational control can depend on protein–protein interaction, based on the low-complexity motifs (LCMs), that indeed keep together different proteins in the already mentioned RNA-containing granules [130].

Among miRNAs, a relationship with spatial memory and synaptic plasticity has been evidenced, for example, in the cases of miR-335-5p [131] and miR-181a [132].

As mentioned above, an important role in mRNA local utilization is also played by mRNA modifications. Thus, enzymes involved in these processes should also be localized. For example, mRNA editing based on deamination of adenosine to inosine is catalyzed by adenosine deaminase RNA-specific (ADAR) enzymes; within this protein family, ADAR3 is highly represented in the brain, especially in some regions, including the hippocampus and amygdala, and it has been found to contribute to mammalian cognitive functions [133]. Interestingly, specific changes in the expression of ADAR enzymes, and hence of the editing events involving the 5-HT2C serotonin receptor (5-HT2CR), have been evidenced in the central amygdala in cases of post-traumatic-stress-disorder (PTSD) [134]. Notably, during neuronal activation, ADAR3 can also transiently translocate to the nucleus [133].

Pre-mRNA splicing is another important step in mRNA function regulation. From this point of view, it is important to emphasize that the Methyl CpG binding domain protein 2 (MeCP2), already known as a DNA methylation “reader”, has been recently found to also regulate alternative splicing events involved in spatial memory consolidation in the mouse hippocampus [135]. Additionally, the use of alternative polyadenylation sites can have an impact on learning and memory events. For this latter reason, some groups have been studying new predictive methods that could allow for the identification of different polyadenylation sites in mRNAs, as well as other modifications, such as different kinds of methylation [136,137,138]. Moreover, some of the alternate splicing portions of mRNA can have a function in localizing it to synapses; for example, it has recently been reported that the 5′UTR derived from exon I is specifically enriched in the BDNF-encoding mRNA targeted to synapses [139].

Among mRNA modifications, an important group is represented by a set of RNA nucleotide modifications, together with their “readers”, that somehow recall the epigenetic DNA modifications (these aspects are probably, again, remnants of the primeval RNA world and have been termed “epitranscriptomics”) [140,141,142,143,144,145,146,147]. One of the most represented mRNA modifications is m6A [147,148] that, when present in the mRNA 5′UTR, is even able to promote CAP-independent translation [141]. Luo et al. have also recently reported a method that allows the identification of a N6,2′-O-dimethyladenosine (m6Am) post-transcriptional mRNA modification [137]. Actually, among m6A-modified RNAs present at synapses, the long noncoding RNA known as Malat1 has also been found; moreover, its synaptic accumulation seems to be a learning-induced event [149].

Interestingly, some years ago it was suggested that the neuronal redox status can have an impact on mRNA methylation and, in turn, on protein synthesis, thus giving further support to the idea that oxidative stress is a basis for neurodegeneration [150].

In addition to mRNAs, ncRNAs have also been found to be modified. Clark et al. have reported, for example, that, during non-associative learning in Aplysia, two transfer RNAs (tRNAs) are highly modified in trained animals; in particular, they found 5-methoxycarbonylmethyl-2-thiouridine (mcm5s2U), and 1-methyladenosine (m1A). These modifications seem to be related to an increase in polyglutamine synthesis [151].

A further important observation concerns the fact that learning and memory also depend on local energetic resources and, hence, on the correct functioning of the synaptic mitochondria; mitochondrial function, in turn, depends on the local synthesis of proteins involved in oxidative phosphorylation (OXPHOS), and it has been recently found that a central role in the synthesis of the nuclear-encoded components of the OXPHOS system is played by the initiation factor eIF4G1. In a mouse model, which was haploinsufficient for this gene, indeed, hippocampal development and memory functions were impaired [152]. When speaking about mitochondria, it is also of interest to remember that prohibitin, a protein of the inner mitochondrial membrane, has been found to allow recovery of learning and memory ability in model mice, after intracerebral hemorrhage, probably by acting on the signaling pathway that involves the Ca^2+^-calmodulin-dependent kinase II (CAMKII) and the collapsin response mediator protein 1 (CRMP1) [153].

Another protein with a local important function is the activity-regulated cytoskeleton-associated protein (Arc) that regulates the local actin cytoskeleton; it also controls the number of membrane glutamate AMPA receptors (AMPARs) in response to neuronal activity [154,155]. Interestingly, specific splicing that involves the 3′UTR region of the Arc mRNA is fundamental for determining a burst of Arc protein production, in response to neuronal activity, and for inducing its involvement in synaptic plasticity [156].

Interestingly, it has been found that many mRNAs that encode proteins involved in memory consolidation have long 3′UTRs; moreover, many of these mRNAs are bound by the growth arrest and DNA damage-inducible protein 45 α (Gadd45), which has been recognized as a regulator of mRNA stability [157].

Finally, it is important to recall that regulation of the learning and memory processes is not only based on neuronal activities. Indeed, it has been clearly demonstrated that glial cells, in particular astrocytes, play fundamental roles. As discussed above, energy availability is necessary for all synaptic activities and especially for cognitive functions. Now, astrocytes have long been known to give metabolic support to neurons not only by simply transferring glucose from the blood–brain barrier to neurons, thanks to the large web they form around the nerve cells, but also because they are able to store glycogen [158,159], which can be used when glucose from the circulation is not immediately available. By breaking down glycogen, and using glucose for glycolysis, they produce lactate, which can then be transferred to neurons through what has been defined as an astrocyte–neuron lactate shuttle (ANLS) [160]. In neurons, lactate can be immediately oxidized to pyruvate and more rapidly used for the tricarboxylic acid cycle [161]. All these metabolic activities of astrocytes have been found to be essential for the highest cognitive functions [162,163]. Moreover, astrocytes contribute to the precise timing of neurotransmission by uptaking neurotransmitters, such as glutamate, from the synaptic cleft.

In addition, they are now known to contribute to neurotransmission because they are able to respond to neurotransmitters and, in particular, to Ca^2+^ signals generated inside the cell, as well as to release their own molecules (gliotransmitters) and, thus, contribute to long-term memory [163,164,165,166,167,168,169]. It has been recently reported that astrocytes also contribute to neuronal excitability and memory formation through the activity of calcineurin (CaN), an enzyme involved in the activation of the astrocytic Na^+^/K^+^ pump [170]. In addition to astrocytes, oligodendrocytes and Schwann cells also have roles in controlling neuronal activity, not only by synthesizing myelin but also because they have been reported to be able to transfer ribosomes to axons, thus allowing translation at very long distances from the neuronal cell body [163,171,172,173].

In general terms, all these glial cell properties require, as in neurons, the ability to transport and localize different species of mRNAs to the periphery, in the vicinity of synapses (for a recent review, see [169]).

Some of these glial cell activities are also mediated by extracellular vesicles (EVs), which are membranous structures that all the cells of the nervous system are able to release and also able to accept from one another [163,174].

### 3.2. Translational Control at the Synapses: Signals and Mechanisms

Neuronal plasticity is the ability to reorganize nervous circuits both during development and aging, as well as, in adults, in response to stimuli coming from the external environment. As discussed above, reorganization of nervous circuits requires modification of synaptic efficacy, which is achieved through both morphological and biochemical rearrangements of the synapses involved. In particular, cognitive functions are based, largely, on long-term variations (Long-term potentiation: LTP) of synaptic regions that require gene activation and synthesis of new proteins, both at pre- and post-synaptic levels [175,176].

While the presence of a protein synthesis system in the dendritic compartment has long been recognized [177], the existence of an analogue process at the axonal periphery has only been more recently accepted on the basis of convincing evidence in favor of the existence of an axonal and presynaptic protein synthesis system [178].

Actually, it has been found that mRNAs present in the nerve endings derive both from the soma of the nerve cells and from a transfer of glial transcripts into the axonal compartment, modulated by glia-axon signaling [179]. Of course, as already discussed, regulation of the translation of these mRNAs is a crucial step for neuronal plasticity. Very often, translation initiation is regulated by phosphorylation of translation initiation factors. In particular, phosphorylation of the eukaryotic translation initiation factor 2alpha (eIF2alpha) plays a central role in memory formation [180]. One of the key factors for the regulation of neuronal plasticity and long-term memory is the mammalian target of the rapamycin (mTOR) signaling pathway [180]. Among the molecules that link synapse activity to local protein synthesis, the already mentioned FMRP and CPEB are of note. It has long been established that FMRP is an mRNA-binding protein associated with polyribosomes and, therefore, implicated in the regulation of protein synthesis. Subsequent studies have shown that it mainly functions as a repressor during the mRNA transport phase; its presence has been primarily detected at dendritic spines, where it regulates protein synthesis at the synapse. As a consequence, in knockout mice, abnormalities in dendritic spines have been observed [181]. The synaptic role of FMRP became clearer when its repressive interaction with the mRNA encoding the metabotropic glutamate receptor mGluR5 was discovered [182].

Actually, FMRP binds mRNAs and other proteins, forming large ribonucleoprotein complexes, which act especially at the level of the post-synaptic vesicles of dendritic spines, inhibiting the translation of mRNAs. Many of the mRNAs bound by FMRP encode proteins involved in synaptic function, and neuronal differentiation, among which are Arc, also known as activity-regulated gene 3.1 (Arg3. 1), αCaMKII, postsynaptic density 95 (PSD-95), synapse-associated protein 90 (SAP90), also known as postsynaptic density protein 95-associated protein 3 (SAPAP3), and microtubule-associated protein 1B (MAP1B) [183,184,185,186,187]. Recent studies have highlighted the involvement of FMRP in the induction of long-term postsynaptic depression (LTD) in response to the activation of group I metabotropic glutamate receptors (mGluR1 and 5). This form of LTD requires the rapid synthesis of proteins at the synapse, which is, in turn, controlled by the inhibitory effect exerted by FMRP on the translation of mRNAs bound to polysomes [188].

Actually, FMRP has been found to regulate mRNA translation by different mechanisms: (i) by masking them in granules [189], (ii) by blocking ribosomal activity [89] and (iii) by inhibiting the elongation factor eIF4E and eIF4G interaction [90]; it can also act in association with the RNA-Induced Silencing Complex (RISC) [190]. However, the role of FMRP in translational regulation is controversial because, in addition to repressing many mRNAs, it can also activate some others [191].

Another protein that is highly important in the regulation of synaptic plasticity is CPEB, which stimulates the translation and elongation of the polyA tail of various messengers [128,192,193,194,195,196]. Richter and Klann [197] have proposed a molecular mechanism according to which CPEB, activated via N-methyl-D-aspartate receptors (NMDAR), stimulates translation of c-jun mRNA; the just-synthesized c-jun protein is transported by a retrograde route to the nucleus where it stimulates Growth Hormone (GH) transcription. Once synthesized, GH is secreted and acts in an autocrine and/or paracrine way, stimulating the strengthening of plasticity, through the activation of the GH receptor. Mediators of these pathways are the phospho-Janus kinase (JAK)J2, the phospho-Signal transducer and the activator of transcription (STAT) 3, which finally enters the nucleus [197].

Aplysia CPEB isoform contains a long stretch of glutamine residues that, as already mentioned, recall those found in prions; indeed, this isoform could take on a prion-like structure upon synaptic stimulation, thus forming a protease-resistant protein at synapses [21,22,198,199]. In vertebrates, there are three genes encoding CPEB-similar proteins; two of these proteins have a polyglutamine sequence but are of shorter length compared to that of Aplysia. Also, in this case, it seems that the sequence of polyglutamine is essential for memory formation [197]. However, it is now known that the Drosophila counterpart, Orb2, and the ApCPEB isoform can be found in the soluble form or in the β-sheet-rich amyloid form, which has greater binding capacity for mRNAs and, although they have low sequence homology, both of them have N-terminal domains that drive aggregation, following synapse activation [128,129,200].

The proposed model has been confirmed many times and involves the formation of liquid-like droplets (LLD) that contain RNAs and proteins; when the synapse is activated, Cap-blocking proteins and deadenylases dissociate and atypical polymerases promote poly(A) tail elongation. These events induce stabilization of the PABP-eiF4G-eiF4E complex, which, in turn, locally activates the translation of CPE-containing mRNAs [128].

Among the mRNAs bound by CPEB, one is that encoding α-CaMKII, which is localized in dendrites and is necessary for synaptic plasticity and LTP; also, in this case, CPEB induces its translational activation by polyadenylation [192].

### 3.3. Inverse Traffic from the Synapse to the Nucleus

As discussed above, synaptic activity induces local expression of new proteins, able to change synaptic structure and strength, thus determining the first modifications related to memory formation. For long-lasting consolidation of memory, however, new transcriptional activity seems to also be necessary [201,202,203]. Thus, we have to envisage the existence of specific signals transferred to the nucleus in both the pre- and the post-synaptic elements. These signals might be given by calcium waves, for example, but also by proteins with nuclear localization sequences (NLS) that, after synthesis at the level of synapses, are transported to the nucleus [204,205]. In reality, however, experimental demonstrations about proteins that function as retrograde messengers for the nucleus are not completely clear (for a recent review, see [203]). Probably, the best known of them is Jacob, a protein highly expressed in the brain cortex, that is able to translocate to the nucleus where it can modulate the activity of the CREB transcription factor [203,206].

Interestingly, it has been reported that some histone protein variants (in particular H2AB) can be downregulated after memory acquisition [207]. This finding is of note because, in addition to transcription factors, histones and, in particular, histone variants, such as H3.3 and H1.0, can have an impact on transcription by regulating the chromatin structure at the level of specific genes [208,209].

In conclusion, RBPs can regulate mRNA translation at the level of synapses, thus allowing for the synthesis of proteins that are able to modify their structure, but perhaps also of proteins able to arrive to the nucleus, thus inducing modifications of transcriptional activity; as a final comment on these properties, we wish to underline that, some years ago, we found that the peptide known as PEP-19 (Purkinje cell expressed peptide)/PCP4 (Purkinje cell protein 4), already known as a Ca^2+^-calmodulin-binding protein, is also able to bind to mRNAs [210]. Moreover, we found that calmodulin, when bound to calcium ions, can compete with mRNAs for binding to PEP-19, although it is not able to bind to RNA on its own [210]. Our observation suggested that the calcium/calmodulin complex, by interacting with PEP-19, may release previously PEP-19-bound mRNAs, thus allowing their translation at the level of synapses at the moment of neurotransmission and, hence, when calcium waves are generated.

### 3.4. Alterations of mRNA and RNA-Binding Protein Prelocalization in Different Pathologies of the Adult Brain

Several neurodegenerative diseases include, among their pathogenetic mechanisms, the altered prelocalization of mRNAs or RBPs or both. As discussed above, mRNA localization in different regions of the cell allows neurons to restrict gene expression to specific products that are quickly available and ready to respond to environmental signals. Prelocalization and local translation could be used to establish inter-neuronal networks on demand and, in this way, sustain synaptic plasticity. There are several mechanisms that can disrupt the pre-localization of mRNA and, in particular, the dysfunction of the proteins responsible for the transport of the mRNA. In several neurodegenerative diseases, such as, for example, amyotrophic lateral sclerosis (ALS) and Alzheimer’s disease (AD), the alteration of mRNA metabolism and dysfunction of RBPs have been documented.

ALS is a neurodegenerative disease whose pathogenesis depends on alterations and mutations of RBPs. One of the main altered RBPs is TAR DNA-binding protein 43 (TDP-43), a protein that plays a major role in mRNA transport by binding to a specific RNA structure, known as a G-quadruplex (G4), a structure also found in the mRNA encoding the amyloid precursor protein (APP) [211]. In neurons, TDP-43, which is ubiquitously expressed in human cells, facilitates the transport of G4-containing mRNAs into neurites. Mutations of TDP-43 are common in ALS and result in its abnormal aggregation in the cytoplasm where it forms the so-called Bunina bodies: ubiquitin-positive and eosinophilic intracellular aggregates [212]. A consequence of this aggregation is a disorder of mRNA axonal transport, due to a destruction of the cytoskeleton, with a subsequent alteration of its normal function in the transport of mRNAs and proteins. Such deficiency affects, in turn, local mRNA localization and translation both in axons and dendrites [213]. TDP-43-induced proteinopathy seems to be mediated by both loss-of-function and gain-of-toxicity mechanisms. Indeed, in ALS, TDP-43 disappears from the nucleus and is localized almost exclusively in the cytoplasm, where its loss-of-function has been shown to cause a reduction in the location of ribosome-encoding mRNAs in axons, thus affecting local translation [214]. Moreover, as shown in a Drosophila model, alteration of the axonal transport mediated by microtubules, with accumulation of TDP-43, causes localization defects of futsch, a protein required for the organization of microtubules at the synapses, as well as of synaptic and dendritic growth [215].

Another protein involved in alterations of mRNA/RBP prelocalization in ALS is the Fused in sarcoma (FUS) RBP. The FUS mutations observed in ALS induce its localization along the axon in the form of aggregates close to the local translation sites of mRNAs. In this way, FUS can affect mRNA metabolism without inducing a loss of its function in the nucleus. Indeed, FUS-proteinopathy has been shown to inhibit intra-axonal protein synthesis in hippocampal neurons and sciatic nerves, thus inducing an integrated stress response. Reduced axonal translation could cause synaptic dysfunction and exacerbations of motor and cognitive symptoms [213].

Notably, mutant FUS has also been reported to affect neuronal chromatin by inducing decompaction and, thus, altered transcription; these effects can also be important for the pathogenic aspects of ALS [216].

AD is the most common form of dementia, and the most common neurodegenerative disease. Pathologically, it is characterized by the formation of extracellular amyloid plaques, and intracellular clusters of tau protein, an axonal microtubule-associated protein, which becomes hyperphosphorylated and aggregates into insoluble complexes. In AD, dysregulation of mRNA metabolism is a pathological hallmark [217]. The evidence that tau regulates mRNA metabolism primarily comes from the fact that tau often colocalizes with many messengers and appears to be capable of performing true functions as an RBP. In fact, tau appeared to be able to interact not only with mRNAs but also with tRNAs and rRNAs, and this could offer an explanation for the reports showing an intranuclear localization of tau. Furthermore, tau, in ways similar to TDP-43, may aggregate with other RBPs to form ribonucleoprotein granules, such as stress granules [218]. As a consequence, translation of mRNA is dysregulated in AD. Polysomes isolated from the brains of AD patients are fewer than in controls, and, in addition, they are endowed with lower translational efficacy only in the brain areas typically affected by AD pathology [219]. Reduced levels of rRNA and tRNA were found in the parietal cortex, but not in the cerebellum, which is in agreement with Langstrom’s findings [220]. However, translation could be influenced by perturbing the mRNA prelocalization process upstream. This, for example, could be hypothesized taking into consideration that it has been shown that, in tau-inducible human embryonic kidney (HEK) cells, tau can upregulate the expression of proteins that contribute to cytoskeleton-dependent axonal transport, thus affecting mRNA localization at axonal and dendritic spine levels, and the tau P301L mutation causes loss of its transcriptional function [221].

As discussed above, mRNA methylation is also important in order to control its post-transcriptional regulation; according to this finding, it has been reported that the m6A methyltransferase 3 (METTL3), and the RNA Binding Motif Protein 15B (RBM15B), a regulator member of the methyltransferase complex (MACOM), are expressed at altered levels in the hippocampus of AD patients [222]; more recently, a further group of RNA methylation regulators, among which ELAV-like RNA binding protein 1 (ELAV1) and the YTH N6-methyladenosine RNA binding protein F2 (YTHDF2), has been found not to be expressed in normal amounts [223].

Similarly, modified levels of RNA methylation have been found in the hippocampus of a mouse model of Hungtington’s disease [224].

In addition to alterations of RBPs and/or of enzymes involved in RNA metabolism, down- or upregulation of microRNAs has also been linked to defective management of post-transcriptional regulation. It has been reported, for example, that downregulation of miR-195, which can repress translation of the mRNAs encoding APP and the β-site amyloid precursor protein cleaving enzyme (BACE1), can also have an impact on dementia in AD; as a consequence, an increase in this miRNA should have a positive effect on AD patients [225,226]. On the other hand, some miRNAs are involved in repressing translation of mRNAs that encode proteins with a fundamental role in learning and memory; in these cases, importance should be placed on finding a way to downregulate them [227,228]. Like in AD, alterations of microRNA expression have also been observed, for example, in Parkinson’s disease (PD) [229], in autism spectrum disorders [230,231] and in psychiatric pathologies [232,233]. Interestingly, impairment of learning and memory processes have also been noted in chronically stressed animals and, in this case, miRNAs also seem to be involved [234].

Thus, in general, given their important role in finely tuning post-transcriptional expression of proteins involved in synaptic plasticity, microRNAs might be central targets in the analysis/therapy of learning and memory processes, neurological diseases and dementia [56,225,235,236,237,238,239,240,241,242,243,244,245,246,247,248].

A further interesting point concerns the observation that sleep deprivation can alter hippocampus-dependent memory by causing alterations at the level of gene transcription, but also by affecting mRNA translation into proteins (for a recent review, see Ref. [249]).

As a final comment, it has been found that some viruses with an RNA genome can interact with proteins of the nervous system. For example, it has been reported that the lymphocytic choriomeningitis virus (LCMV) can affect expression of the GAP-43 protein, both at the transcriptional and post-transcriptional level; thus, given the already mentioned importance of this protein as a coordinator of proteins involved in axonal structure and function, the effect of this virus is deleterious for neuronal plasticity [250]. Similarly, the RNA genome of the tick-borne encephalitis virus (TBEV) is transported to dendrites, where it is also replicated; for the transport, it is loaded into granules by interacting with RBPs that should work for the localization of endogenous mRNAs. As a result, normal transport of mRNA in infected neurons is altered, giving rise to neurological disorders [251]. Actually, it is now clear that a great number of RBPs are able to interact with viral RNA [252], and some of them can have a role in controlling infection; however, we can envisage that these interactions could also interfere with the physiological functions of RBPs, a problem with a potentially high impact on the nervous system.

## 4. Conclusions and Perspectives

In conclusion, post-transcriptional regulation of mRNA maturation, subcellular localization and translation is fundamental for a correct development of the nervous system, as well as for all the functions of the adult brain, including the highest ones, such as learning and memory. Given the involvement in this regulation of RBPs, RNA-modifying enzymes and ncRNAs, it is of the utmost importance to acquire as much information as possible regarding these molecules. We can, indeed, envisage that many neurological pathologies might be, at least in part, corrected by acting on a number of the mentioned regulatory molecules, which are normally produced by neurons. In particular, given the ability of all the brain cells to produce and receive extracellular vesicles (EVs), we can hope that, in the near future, we will be able to load EVs with the necessary molecules, a number of which are altered in diseased neurons, and to deliver them to the nervous system; indeed, EVs are able to cross the blood–brain barrier and might reach the brain cells, especially if we are able to equip them with membrane proteins specifically recognizable by the receptors present on the target cells.
